# 

**Published:** 1872-08

**Authors:** 


### CONTENTS:

ORIGINAL COMMUNICATIONS:

ARTICLE I. Disease Germs. By I. N. Danforth, M.D., Chicago. Second paper.......... 449

ART. II. Clinical Lectures on Diseases of the Ear. By E. L. Holmes, M.D., Chicago.......... 454

ART. III. Notes from the Rear Rank. By G. Newkirk, M.D., New Boston, Mo.......... 457

SELECTIONS:

On Catarrh of the Uterus.......... 460

A Plea for the Antiphlogistic Treatment of Disease.......... 474

Removal of a large Fibro-Cystic Tumor of the Uterus by Gastrotomy.......... 489

Blood Stains.......... 490

EDITORS' BOOK TABLE.......... 497

EDITORIAL.......... 499

LOOT.......... 504

NOTICES.......... 511

